# Electromechanical Modeling and Dimensionless Optimization of a Multilayer PZT-5H Energy Harvester for Vehicle Suspension Systems

**DOI:** 10.3390/mi17101111

**Published:** 2026-09-23

**Authors:** Swati Devi, Vijay Kumar Gupta, Ivan A. Parinov

**Affiliations:** 1 MDV Lab, Mechanical Engineering Discipline, PDPM Indian Institute of Information Technology, Design and Manufacturing, Jabalpur 482005, Madhya Pradesh, India; vkgupta@iiitdmj.ac.in; 2 I. I. Vorovich Mathematics, Mechanics and Computer Sciences Institute, Southern Federal University, Rostov-on-Don 344090, Russia; parinov_ia@mail.ru

**Keywords:** vehicle suspension, piezoelectric energy harvesting, multilayer PZT-5H, electromechanical modeling, dimensionless analysis, Taguchi-ANOVA optimization

## Abstract

Vehicle suspension systems dissipate substantial vibration energy, generated by tyre–road interactions, most of which is lost as heat through damping. Recovering this energy can provide a sustainable power source for self-powered sensing and intelligent vehicle monitoring. This study presents a coupled electromechanical model of a quarter-car suspension incorporating a multilayer PZT-5H stack in series configuration for vibration energy harvesting. The proposed arrangement preserves effective suspension stiffness while enhancing electrical output through cumulative voltage generation. The governing mechanical and electrical equations are reformulated into a dimensionless framework, enabling generalized evaluation of mass ratio, stiffness ratio, damping, electromechanical coupling, and electrical load. The analytical model is independently verified using a MATLAB-Simulink (R2025b) implementation, while available experimental data are used only as an external benchmark rather than as direct full-scale validation. Parametric analysis shows that harvested power peaks near the suspension-bounce resonance, while stiffness ratio and mass ratio significantly influence strain transfer and inertial energy exchange, respectively. Taguchi-based design of experiments and ANOVA reveal that load resistance is the dominant factor, contributing approximately 63.6% and 58.5% of the variance for the undamped and damped models, respectively, followed by stiffness ratio (19.6% and 17.9%) and mass ratio (14.0% and 12.5%). The proposed framework integrates the series-connected piezoelectric configuration, dimensionless modeling, analytical–numerical verification, and statistical optimization to provide a unified methodology for designing suspension-integrated piezoelectric energy harvesters.

## 1. Introduction

Accurate measurement and prediction of vibration-induced responses in vehicle suspension systems are essential for enhancing comfort, structural health monitoring, intelligent transportation, and self-powered sensing. During vehicle operation, a significant portion of vibration energy, generated through tyre–road interactions, is dissipated as heat by suspension dampers. Therefore, recovering this wasted energy through vibration energy harvesting has attracted significant research interest as a means of powering low-power sensors, wireless monitoring devices, and intelligent electronic systems while reducing battery dependence [1,2,3,4,5,6,7]. Among the available energy-harvesting technologies, including electromagnetic, electrostatic, triboelectric, and piezoelectric approaches, piezoelectric energy harvesting has emerged as one of the most promising solutions, owing to its high energy density, compact size, fast electromechanical response, and suitability for the low-frequency excitation encountered in vehicle suspensions [2,3,4,5,6,7]. Advances in multilayer PZT stacks, operating on the $d_{33}$ mode, exhibit a higher blocking force, superior electromechanical coupling, and higher voltage sensitivity, making them promising candidates for suspension-integrated vibration energy-harvesting applications.

Considerable research has been devoted to improving suspension-based energy harvesting. Experimental and numerical studies have demonstrated the feasibility of integrating piezoelectric transducers into vehicle shock absorbers, regenerative suspensions, and hybrid energy-harvesting systems, confirming their potential for recovering vibration energy under realistic operating conditions [8,9,10,11,12,13,14]. In parallel, advances in vehicle dynamics, suspension control, and road-induced vibration analysis have improved understanding of vibration transmission characteristics and the amount of recoverable energy available in automotive suspension systems [15,16,17].

To enhance energy-harvesting performance over a broad range of operating conditions, researchers have proposed tuned auxiliary structures, multimodal and two-degree-of-freedom harvesters, nonlinear mechanisms, broadband configurations, and impedance-matching techniques [18,19,20,21,22,23]. While these approaches have significantly improved the harvested power and operational bandwidth, many introduce additional stiffness, damping, or mechanical complexity that can modify the dynamic characteristics of the suspension system. Maintaining an appropriate balance between energy-harvesting efficiency and vehicle dynamic performance remains a major challenge in the practical design of suspension-integrated piezoelectric energy harvesters.

In a conventional parallel-connected piezoelectric suspension configuration, the piezoelectric element acts as an additional load-bearing stiffness in parallel with the suspension spring. This increases the effective suspension stiffness and can shift the suspension natural frequencies, thereby potentially altering the vehicle dynamic response and ride-comfort characteristics. In contrast, a series-connected PZT stack introduces the piezoelectric element along the suspension force path while maintaining an effective stiffness close to that of the original suspension when the PZT stiffness is substantially higher than the suspension spring stiffness. Therefore, the motivation for the series configuration is not the novelty of the electrical connection itself, but its ability to enable energy harvesting while minimizing modification of the baseline suspension stiffness.

Analytical modeling plays an important role in understanding the coupled interactions between vehicle suspension dynamics and electrical power generation, thereby providing the theoretical basis for the design and optimization of suspension-integrated energy harvesters. Previous studies have employed impedance-based analyses and dimensionless formulations to investigate the electromechanical behavior of piezoelectric energy harvesters and the influence of key design parameters [23,24]. In addition, statistical optimization techniques, particularly the Taguchi method and analysis of variance (ANOVA), have been widely adopted to identify the most influential design variables [25,26]. To validate the results and to ensure the reliability of analytical and numerical modeling approaches, researchers also performed experiments [27,28].

Recent studies have investigated data-driven optimization strategies and multidirectional piezoelectric harvesters for improving vibration energy-harvesting performance [29,30]. Further attention has been directed towards electrical efficiency, emerging harvesting technologies, and intelligent vehicle suspension systems [31,32,33,34,35]. Consequently, a comprehensive methodology that simultaneously combines analytical modeling, independent numerical verification, and systematic statistical optimization remains insufficiently explored.

To overcome these limitations, the present study develops a unified electromechanical modeling and optimization framework for a quarter-car suspension incorporating a series-connected multilayer PZT-5H stack. Unlike conventional parallel-connected configurations, the proposed series-connected arrangement preserves the effective suspension stiffness while enhancing the output voltage through cumulative electrical generation from the multilayer piezoelectric stack. Following numerical verification, the proposed framework is integrated with the Taguchi design of experiments and analysis of variance (ANOVA) to identify the dominant dimensionless parameters governing suspension response, generated voltage, harvested power, and electromechanical conversion efficiency.

Series-connected piezoelectric stacks integrated with vehicle suspension springs have been previously reported [11]. Therefore, the series electrical/mechanical arrangement itself is not claimed as a standalone novelty in the present work. Instead, the novelty lies in the explicit incorporation of the series-connected multilayer PZT-5H stack, including its stiffness and intermediate deformation co-ordinate, into a fully coupled quarter-car electromechanical formulation, followed by dimensionless analysis, independent analytical–numerical verification, and Taguchi-ANOVA-based parameter analysis.

The objective of this study is to quantify how the governing dimensionless mechanical and electrical practical parameters affect suspension response and harvesting performance, and to identify their relative importance through independent numerical verification and Taguchi-ANOVA analysis.

The principal contributions of this work are four-fold: (i) the development of a series-connected multilayer PZT-5H suspension energy harvester incorporated into a fully coupled quarter-car electromechanical model, with explicit consideration of the PZT-stack stiffness and intermediate deformation co-ordinate; (ii) the formulation of a fully coupled dimensionless electromechanical model for performance prediction; (iii) independent analytical-numerical verification of the proposed model; and (iv) the integration of Taguchi-ANOVA-based statistical optimization to identify the governing design parameters. Collectively, these contributions provide a unified framework for the design, performance prediction, and optimization of suspension-integrated piezoelectric energy harvesters for intelligent vehicle monitoring and self-powered sensing applications.

The remainder of this paper is organized as follows. Section 2 presents the mathematical formulation of the quarter-car suspension system, the proposed series-connected piezoelectric configuration, the coupled electromechanical model, and its dimensionless representation. Section 3 describes the analytical implementation and the corresponding Simulink model, used for independent numerical verification. Section 4 introduces the Taguchi-ANOVA-based optimization methodology for evaluating the influence of the governing dimensionless parameters. Section 5 presents and discusses analytical, numerical, parametric, and statistical results. Finally, Section 6 summarizes the principal findings, conclusions, and potential directions for future research.

## 2. Mathematical Model of Quarter-Car

The proposed model integrates the dimensionless electromechanical formulation of Xiao et al. [24] with a series-connected multilayer PZT stack, integrated into a quarter-car suspension. This formulation establishes the coupled mechanical-electrical governing equations required to predict dynamic response, voltage generated, and power harvested under harmonic excitation.

### 2.1. Mechanical Model of the Quarter-Car System Without PZT Stack

The quarter-car (QC) model is adopted to describe the vertical dynamics of the vehicle suspension because it accurately represents bounce and wheel-hop motions while maintaining low computational complexity. Owing to its simplicity and effectiveness, it has been extensively employed in studies of suspension dynamics, ride-comfort evolution, and suspension-based vibration energy harvesting [9,10,11,12,13,33].

As shown in Figure 1a,b, the system consists of the sprung mass ($M_{s}$), unsprung mass ($M_{us}$), suspension spring ($K_{s}$), suspension damper ($C_{s}$), tyre stiffness ($K_{s}$), tyre damping ($C_{us}$), and the road-displacement input ($Z_{R}$). The model assumes linear spring and viscous damping characteristics, small vertical vibrations, rigid mass, continuous tyre-road contact, and time-invariant system parameters.

Applying Newton’s second law to the free-body diagram yields the governing equations of motion:

Sprung mass

(1) $$M_{s}{\ddot{Z}}_{s}=-C_{s}\left({\dot{Z}}_{s}-{\dot{Z}}_{us}\right)-K_{s}\left(Z_{s}-Z_{us}\right)$$

Unsprung mass

(2) $$M_{us}{\ddot{Z}}_{us}=C_{s}\left({\dot{Z}}_{s}-{\dot{Z}}_{us}\right)+K_{s}\left(Z_{s}-Z_{us}\right)-C_{us}\left({\dot{Z}}_{us}-{\dot{Z}}_{R}\right)-K_{us}\left(Z_{us}-Z_{R}\right)$$

where $Z_{s},\;Z_{us}$, and $Z_{R}$ denote the vertical displacements of the sprung mass, unsprung mass, and road surface, respectively, while the overdot represents differentiation with respect to time.

For frequency-domain analysis, zero initial conditions are assumed, and the equations are transformed into the Laplace domain.

The standard quarter-car frequency-domain transformations are well established in the literature [9,10,11,23]; therefore, only the relations required for the present series-connected configuration are retained. The displacement transfer functions of the sprung and unsprung masses are subsequently obtained as

(3) $$G_{s}\left(s\right)=\frac{Z_{s}\left(s\right)}{Z_{R}\left(s\right)},G_{us}\left(s\right)=\frac{Z_{us}\left(s\right)}{Z_{R}\left(s\right)}$$

These transfer functions provide the basis for evaluating the dynamic response and resonance characteristics of the suspension system and are widely employed in vehicle dynamics and vibration energy-harvesting analyses [10,11,23,24]. For harmonic road excitation, $Z_{R}(t)=Z_{0}e^{j\omega t}$ is assumed.

The frequency-domain relations ${\dot{Z}}_{R}=\;$*s*$Z_{R}$, and ${\ddot{Z}}_{R}=s^{2}Z_{R}$ are directly applied to determine the steady-state response. The developed mechanical model is subsequently extended by incorporating the series-connected multilayer piezoelectric stack to establish the coupled electromechanical formulation presented in Section 2.2.

### 2.2. Piezoelectric-Stack Configuration and Equivalent Electrical Representations

A multilayer piezoelectric (PZT) stack operating on the longitudinal $d_{33}$ mode is adopted because it provides high electromechanical coupling, high blocking force, and efficient energy conversion under compressive loading, making it suitable for vehicle suspension applications [2,11,23,27,30]. Unlike cantilever-based harvesters, multilayer PZT stacks can sustain large compressive forces while producing higher electrical output in compact configurations. In the proposed system, the PZT stack is mechanically arranged in series with the suspension spring, i.e., it is inserted along the suspension force path between the sprung and unsprung masses. This mechanical series arrangement should be distinguished from the electrical interconnection of individual piezoelectric layers to preserve the original suspension characteristics while enabling efficient mechanical strain transfer, as illustrated in Figure 2a.

The analytical model assumes linear piezoelectric behavior, uniform axial stress, one-dimensional deformation, negligible electrode thickness, and operation under the small-displacement regime. The piezoelectric stack is further assumed to operate in the $d_{33}$ mode without hysteresis or dielectric losses, consistent with commonly adopted electromechanical modeling approaches [23,24,27].

The principal advantage of the proposed configuration is that the suspension stiffness remains practically unchanged. For the series and parallel-connection configuration shown in Figure 2a,b, the equivalent stiffness is

(4) $$K_{eq}^{series}=\frac{K_{p}K_{s}}{K_{p}+K_{s}},\;K_{eq}^{parallel}=K_{s}+K_{p}$$

where $K_{s}$ and $K_{p}$ denote the suspension spring and piezoelectric-stack stiffness, respectively. Since ($K_{p}\gg K_{s}$) the adopted PZT stack has a stiffness much higher than that of the suspension spring, the equivalent series stiffness reduces to

(5) $$K_{eq}^{series}\approx K_{s}$$

Thus, the series-connected stack contributes negligible additional stiffness to the baseline suspension, whereas the parallel configuration increases the effective stiffness. This provides the mechanical basis for adopting the series arrangement in the proposed harvester.

(6) $$F_{p}=K_{p}(Z_{s}-Z_{p})+\theta V_{p}(t)$$

where $F_{p}$ is the piezoelectric force, $Z_{p}$ is the stack displacement, $V_{p}$ is the generated voltage, and θ is the electromechanical force factor. The electromechanical response follows charge conservation and is governed by

(7) $$I_{p}=\theta {\dot{(Z}}_{s}-{\dot{Z}}_{p})-C_{p}{\dot{V}}_{p}$$

where $I_{p}$ and $C_{p}$ denote the generated current and the blocked capacitance of the multilayer PZT stack, respectively [23,24,27].

The multilayer PZT stack is represented by the equivalent electrical circuit shown in Figure 2c, comprising a deformation-dependent current source $\theta {\dot{Z}}_{p}$, intrinsic capacitance $C_{p}$, and leakage resistance $R_{p}$. The current source models the electrical charge generated by the direct piezoelectric effect, whereas $C_{p}$ represents the inherent dielectric capacitance of the stack. Since leakage resistance is several orders of magnitude larger than the external load resistance, its effect on the harvested power is neglected.

The generated AC voltage is converted into a stable DC output using a full-wave bridge rectifier and a smoothing capacitor $C_{L}$, which minimizes voltage ripple across the external load resistance $R_{L}$. Accordingly, the harvested output power is calculated as

(8) $$P=\frac{V_{out}^{2}}{R_{L}}$$

where $V_{out}$ is the rectified DC voltage across the load. The equivalent electrical model is subsequently coupled with a mechanical quarter-car model to establish the complete electromechanical formulation for predicting the harvested voltage and power.

### 2.3. Electromechanical QCM Model

The mechanical quarter-car system is coupled with the piezoelectric relations presented in Section 2.2. As shown in Figure 1c,d, the piezoelectric stack is positioned in series with the suspension spring, with the corresponding free-body diagram shown in Figure 1d. The resulting model includes the sprung-mass displacement $Z_{s}$, unsprung-mass displacement $Z_{us}$, and intermediate piezoelectric-stack co-ordinate $Z_{p}$. The electromechanical interaction is represented by the force factor θ, which couples the mechanical and electrical responses [19,23,24,27].

The dynamic equilibrium of the sprung mass is expressed as

(9) $$M_{s}{\ddot{Z}}_{s}+C_{s}\left({\dot{Z}}_{s}-{\dot{Z}}_{us}\right)+K_{p}\left(Z_{s}-Z_{p}\right)+\theta V=0$$

The unsprung-mass equation is

(10) $$\;\;M_{us}{\ddot{Z}}_{us}+C_{s}\left({\dot{Z}}_{us}-{\dot{Z}}_{s}\right)+K_{s}\left(Z_{us}-Z_{p}\right)+C_{us}\left({\dot{Z}}_{us}-{\dot{Z}}_{R}\right)+K_{us}\left(Z_{us}-Z_{R}\right)=0$$

The dynamic equilibrium of the piezoelectric-stack co-ordinate is

(11) $$M_{p}{\ddot{Z}}_{p}+K_{p}\left(Z_{p}-Z_{s}\right)+K_{s}\left(Z_{p}-Z_{us}\right)-\theta V=0$$

The electrical-domain equations, obtained from charge conservation, are

(12) $$\dot{V}+\frac{1}{C}\left[\theta \left({\dot{Z}}_{p}-{\dot{Z}}_{s}\right)+\frac{V}{R}\right]=0$$

Standard intermediate transformations have been omitted because they are established in [23,24]. The governing equations specific to the proposed series-connected PZT configuration are retained, and the intermediate transformed relations are provided in Appendix A.1.

(13) $$\;\;\;\;\;\;\left[RC_{p}M_{s}s^{3}+RC_{p}C_{s}s^{2}+RC_{p}K_{p}s+M_{s}s^{2}+C_{s}s+K_{p}+\theta^{2}Rs\right]Z_{s}-\left[RC_{p}C_{s}s^{2}+C_{s}s\right]Z_{us}\;-\left[RC_{p}K_{p}s+K_{p}+\theta^{2}Rs\right]Z_{p}=0$$

Similarly, substitution into the piezoelectric-stack equation gives

(14) $$-\left[RC_{p}K_{p}s+K_{p}+\theta^{2}Rs\right]Z_{s}-\left[RC_{p}K_{p}s+K_{s}\right]Z_{us}\;\;\;\;\;\;\;\;\;\;\;\;\;\;\;\;\;+\left[RC_{p}M_{p}s^{3}+RC_{p}K_{p}s+RC_{p}K_{s}s+M_{p}s^{2}+K_{p}+K_{s}+\theta^{2}Rs\right]Z_{p}=0$$

The corresponding unsprung-mass relation remains

(15) $$-C_{s}sZ_{s}+\left[M_{us}s^{2}+C_{s}s+C_{us}s+K_{s}+K_{us}\right]Z_{us}-K_{s}Z_{p}=\left[C_{us}s+K_{us}\right]Z_{R}$$

The coupled frequency-domain response is obtained by simultaneously solving Equations (13)–(15) for $Z_{s}$, $Z_{us},$ and $Z_{p}$ at a prescribed road excitation $Z_{R}$.

For harmonic road excitation, the displacement responses are represented by their complex steady-state amplitudes at the excitation frequency ω.

The generated voltage is consequently obtained by simplifying the coupled frequency-domain relations and substituting the resulting displacement responses into the piezoelectric voltage relation in Equation (16).

(16) $$V\left(j\omega \right)=\frac{j\omega \theta R[Z_{s}\left(j\omega \right)-Z_{p}(j\omega )]}{\sqrt{1+j\omega^{2}R^{2}C_{p}^{2}}}$$

The voltage amplitude is obtained from the magnitude of the complex voltage in Equation (16).

The voltage response may be normalized with respect to the base-acceleration amplitude for comparison across excitation levels.

For harmonic steady-state operation, Equation (16) gives the complex output voltage phasor, whose magnitude represents the peak voltage amplitude. As shown in Figure 2c, under the ideal full-wave rectifier and smoothing-capacitor assumption, the rectified load voltage is represented by this peak magnitude; hence, the corresponding maximum rectified load power is

(17) $$P_{out}\left(\omega \right)=\frac{{\left|V(j\omega )\right|}^{2}}{2R}$$

The time-average mechanical input power associated with the harmonic road excitation is obtained from the transmitted force and road velocity as

(18) $$P_{in}\left(\omega \right)=\frac{1}{2}R_{e}\{\left(K_{us}+j\omega C_{us}\right)[Z_{R}\left(j\omega \right)-Z_{us}\left(j\omega \right)]\left[j\omega Z_{R}\left(j\omega \right)\right]*\}$$

The electromechanical energy-conversion efficiency is finally defined as

(19) $$\eta \left(\omega \right)=\frac{\frac{\omega^{2}\theta^{2}R{\left|Z_{s}\left(j\omega \right)-Z_{p}\left(j\omega \right)\right|}^{2}}{2({1+\omega }^{2}R^{2}C_{p}^{2})}}{\frac{1}{2}R_{e}\{\left(K_{us}+j\omega C_{us}\right)[Z_{R}\left(j\omega \right)-Z_{us}\left(j\omega \right)]\left[j\omega Z_{R}\left(j\omega \right)\right]*\}}$$

Equations (13)–(15) provide the coupled mechanical response of the suspension-piezoelectric system, while Equations (16), (17) and (19) determine the generated voltage, harvested electrical power, and electromechanical conversion efficiency, respectively. These equations are from the frequency-domain analytical model used for comparison with the corresponding time-domain Simulink implementation. The superscript “*” denotes the complex conjugate of a frequency-domain quantity.

### 2.4. Dimensionless Formulation of the Coupled Electromechanical Model

The coupled equations are nondimensionalized to separate the effects of inertia, stiffness, damping, electromechanical coupling, and electrical loading from the absolute system scale, following the formulation of Xiao et al. [24]. The intermediate PZT co-ordinate is retained to describe deformation transfer through the series-connected stack.

The suspension natural frequency, dimensionless time, and normalized excitation frequency are defined as

(20) $$\omega_{s}=\sqrt{\frac{K_{s}}{M_{s}}},\;\tau =\omega_{s}t,\Omega =\frac{\omega }{\omega_{s}}$$

We shall use the road-displacement amplitude $Z_{0}$ as the reference length,

(21) $$x_{s}=\frac{Z_{s}}{Z_{0}},\;x_{us}=\frac{Z_{us}}{Z_{0}},\;x_{p}=\frac{Z_{p}}{Z_{0}},x_{R}=\frac{Z_{R}}{Z_{0}},$$

The dimensionless mechanical parameters are defined as

(22) $$M_{r}=\frac{M_{us}}{M_{s}},\;M_{pr}=\frac{M_{p}}{M_{s}},K_{r}=\frac{K_{us}}{K_{s}},K_{pr}=\frac{K_{p}}{K_{s}},\;C_{r}=\frac{C_{s}}{M_{s}\omega_{s}},\;C_{ur}=\frac{C_{us}}{M_{s}\omega_{s}}$$

The dimensionless voltage, electromechanical coupling, and electrical load are defined as

(23) $$\upsilon =\frac{\theta V}{K_{s}Z_{0}},K^{2}=\frac{\theta^{2}}{K_{s}C_{p}},R*=\omega_{s}RC_{p}$$

Here, $K_{pr}$, characterizes the stiffness of the series-connected PZT stack relative to the suspension spring, $K^{2}$ represents the normalized electromechanical coupling strength, and R* compares the electrical RC time scale with the characteristic mechanical time scale. The adopted scaling is consistent with dimensionless and impedance-based piezoelectric energy-harvesting formulations [19,23,24,27].

The dimensionless equations of Section 2.3 are reduced to

(24) $$x_{s}^{\prime \prime }+C_{r}\left(x_{s}^{\prime }-x_{us}^{\prime }\right)+K_{pr}\left(x_{s}-x_{p}\right)+\upsilon =0$$

(25) $$M_{r}x_{us}^{\prime \prime }+C_{r}\left(x_{us}^{\prime }-x_{s}^{\prime }\right)+C_{ur}\left(x_{us}^{\prime }-x_{R}^{\prime }\right)+\left(x_{us}-x_{p}\right)+K_{r}\left(x_{us}-x_{R}\right)=0$$

(26) $$M_{pr}x_{p}^{\prime \prime }+\left(x_{p}-x_{us}\right)+K_{pr}\left(x_{p}-x_{s}\right)-\upsilon =0$$

and

(27) $$\upsilon^{\prime }+\frac{\upsilon }{R*}-K^{2}\left(x_{s}^{\prime }-x_{p}^{\prime }\right)=0$$

where the prime denotes differentiation with respect to τ.

Equations (24)–(27) constitute the dimensionless electromechanical model of the proposed harvester. The terms $K_{pr}\left(x_{p}-x_{s}\right)$ and $\left(x_{p}-x_{us}\right)$ explicitly preserve the mechanical force-transfer path through the series-connected suspension-PZT arrangement, while $\left(x_{s}^{\prime }-x_{p}^{\prime }\right)$ represents the relative deformation rate governing electrical generation. Thus, the PZT stack is retained as an electromechanically coupled intermediate element rather than being replaced by an equivalent parallel stiffness.

Hence, the dimensionless generated voltage is

(28) $$V^{*}=\frac{j\Omega K^{2}R*}{1+j\Omega R*}\left(X_{s}-X_{p}\right)$$

Equation (28) directly relates the electrical output to the relative deformation of the series-connected PZT stack, electromechanical coupling, excitation frequency, and normalized electrical load.

Substituting Equation (28) into the rectified load-power expression (Equation (17)) and normalizing by the reference power, the dimensionless harvested power is obtained as

(29) $$P_{out}^{*}=\frac{\Omega^{2}K^{2}R*}{2\left(1+\Omega^{2}R^{*2}\right)}{\left|X_{s}-X_{p}\right|}^{2}$$

Equation (29) is the principal dimensionless power relation used in the subsequent parametric and optimization analyses. It separates the mechanical deformation contribution ${\left|X_{s}-X_{p}\right|}^{2}$ from the electromechanical coupling and electrical load effects. For a fixed mechanical response, the electrical load term is maximized when

(30) $$R_{opt}^{*}=\frac{1}{\Omega }$$

which represents the resistive impedance-matching condition and explains the rise-peak-drop variation of harvested power with load resistance [23,24,27].

The dimensionless harvested power is normalized by the reference power $P_{0}$ = $K_{s}Z_{R}^{2}\omega_{s}$. Using reference parameters listed in Table 1 and excitation specified in Section 3, $Z_{R}$= 0.118 m and $\omega_{s}$ = 10 rad/s, giving $P_{0}=3.63\;kW.$ The dimensionless formulation is used for parameter-sensitivity analysis, while dimensional quantities are retained for practical performance evaluation.

(31) $$\eta =\frac{\frac{\Omega^{2}K^{2}R*}{\left(1+\Omega^{2}R^{*2}\right)}{\left|X_{s}-X_{p}\right|}^{2}}{R_{e}\{\left(K_{r}+{j\Omega C}_{us}\right)\left(1-X_{us}\right)\left(-j\omega \right)\}}$$

Equations (28), (29) and (31) define the dimensionless voltage, harvested power, and conversion efficiency, used in the subsequent analyses. The formulation retains the intermediate PZT co-ordinate and therefore explicitly captures the relative deformation ($X_{s}-X_{p}$) of the mechanically series-connected stack. The resulting response is governed by $M_{r},M_{pr},K_{r},K_{pr},C_{r},C_{ur},K^{2},R*,\;\mathrm{and}\;\Omega$ providing the scale-independent basis for the parametric and Taguchi-ANOVA analyses presented in the following sections.

## 3. Quarter-Car Suspension Model and Simulation

The coupled electromechanical formulation was implemented independently in MATLAB and Simulink using identical mechanical, piezoelectric, and electrical parameters. The analytical frequency-domain model provides a steady-state response, while the Simulink model provides numerical representation of the same electromechanical system. This modeling approach enables direct verification of the predicted displacement, voltage, and harvested-power responses without altering the proposed system-level formulation.

The model parameters used throughout the study are listed in Table 1. The mechanical properties were adopted from representative quarter-car suspension models, whereas the piezoelectric parameters correspond to the multilayer PZT stack employed in the proposed harvester [9,10,11,23,24,33]. The same parameter set was maintained in both analytical and numerical models to ensure a consistent comparison.

An external experimental benchmark is provided by Darabseh et al. [11], who tested a small-scale quarter-car suspension with $M_{s}=1\;kg$, $M_{us}=0.6\;kg$, $K_{s}=6200\;N/m$, $K_{us}=2800\;N/m$, and 50 Ns/m suspension damping. Their PZT stack had a mass of 15 g, length of 40 mm, area of 49 mm^2^, capacitance of 4.5 μF, and $d_{33}=600\;pm/V$. At 0.05 g excitation, approximately 0.12 V and 0.36 mW were experimentally reported near resonance. These data provide an external experimental benchmark, while direct one-to-one numerical comparison is not expected because the present study uses representative full-scale vehicle parameters.

Figure 3 presents the Simulink implementation of the conventional and PZT-integrated suspension models. The proposed model includes the intermediate PZT co-ordinate and electromechanical coupling; corresponding analytical and numerical results are compared in Section 5.2.

A harmonic road excitation corresponding to a peak base acceleration of 1 g was applied at the fundamental suspension bounce of 1.45 Hz, selected to examine the response at the primary suspension-bounce resonance [9,10,11,23]. The corresponding road-displacement amplitude was calculated as

(32) $$Z_{0}=\frac{9.81}{{\left(2\pi f\right)}^{2}}$$

which gives $Z_{R}\approx 0.118$ m for f=1.45 Hz. This acceleration level is retained as a conservative, bounding excitation case used consistently for model verification and parametric comparison, rather than as a claim of typical in-service road roughness; the correspondingly large displacement amplitude follows from evaluating the response at the low-frequency suspension-bounce mode and should be interpreted accordingly. The same excitation, electrical load, and model parameters were applied in both MATLAB and Simulink to ensure a consistent numerical verification framework. After numerical verification, the analytical and Simulink models were used for the subsequent frequency-response, parametric, and Taguchi-ANOVA analysis.

## 4. Parameter Optimization

The numerically verified analytical and Simulink models developed in Section 2 and Section 3 served as the computational framework for generating the response dataset used in the subsequent optimization. To identify the dominant design parameters and determine robust operating conditions without performing a deterministic and exhaustive parametric search, a Taguchi design of experiments (DOE) combined with analysis of variance (ANOVA) was employed as a statistical post-processing technique for the simulation-generated dataset. This approach has been successfully applied to simulation-based optimization of piezoelectric energy harvesters and vibration systems owing to its computational efficiency and capability to quantify parameter significance [25,26,27,33].

The optimization was performed independently for the undamped and damped suspension models using the dimensionless harvested power (Equation (29)) as the performance index. Figure 4 illustrates the adopted optimization workflow, whereas Table 2 and Table 3 summarize the Taguchi response analysis and corresponding ANOVA results, respectively. The detailed orthogonal arrays employed in the optimization are provided in Appendix A.2.

### 4.1. Taguchi Orthogonal Array Selection

Four dimensionless control factors for the undamped model and five factors for the damped model were investigated at three levels, as summarized in Table 2. A conventional full-factorial design would require 3^4^ = 81 and 3^5^ = 243 simulation cases, respectively. To reduce computational effort while preserving balanced estimation of the main effects, the Taguchi L27 orthogonal array was adopted [25,26], reducing the required analyses to 27 simulation cases for each model.

#### Signal-to-Noise (S/N) Ratio and ANOVA

Since the objective was to maximize harvested power, the Larger-the-better (LTB) signal-to-noise (S/N) criterion was adopted [25,26]:

(33) SNLTB=−10log101n∑i=1n1Pi2

where $P_{i}$ denotes the dimensionless harvested power obtained from the numerically verified analytical and Simulink models for the $i^{th}$ design point.

The resulting S/N responses are summarized in Table 2, where the effect range (∆) and the ranking provide an initial measure of the relative influence of each design parameter. Table 3 presents the corresponding ANOVA results, including the F-statistic and percentage contribution, which quantify the statistical significance of each parameter. Together, these analyses identify the dominant dimensionless variables governing the harvested power of the proposed suspension energy harvester.

Statistical significance was assessed at the 95% confidence level, with *p* < 0.05 considered statistically significant. For the undamped model, load resistance, stiffness ratio, and mass ratio were statistically significant (*p* < 0.001), whereas the electromechanical coupling factor was not significant (*p* = 0.993). For the damped model, load resistance, stiffness ratio, mass ratio, and electromechanical coupling factor were statistically significant (*p* < 0.001), whereas the damping ratio was not significant (*p* = 0.985). The residual contributions are 2.71% and 4.63% for the undamped and damped models, respectively, indicating that the selected factors explain the majority of the variation in the simulation-generated dataset.

The residual terms represent the unexplained variation in the simulation-generated ANOVA dataset; no formal residual-assumption diagnostics or experimental residual validation are claimed.

## 5. Results and Discussion

The results are presented below in terms of parametric response and statistical significance. The performance of the proposed series-connected multilayer piezoelectric-stack energy harvester was investigated through analytical frequency-domain simulations and independently verified using the corresponding Simulink implementation. The analysis focused on understanding the influence of the dimensionless mechanical and electrical parameters on voltage generation, harvested power, and electromechanical conversion efficiency. Subsequently, the relative importance of the governing parameters was quantified using the Taguchi-ANOVA methodology.

### 5.1. Effect of Dimensionless Mechanical Parameters

Figure 5 illustrates the influence of the stiffness ratio and mass ratio on the normalized harvested power and electromechanical conversion efficiency. A quantitative power-efficiency trade-off is observed as stiffness ratio increases. Increasing the stiffness ratio from 0.2 to 0.73 raises normalized harvested power from approximately 0.06 to 0.25, corresponding to a 4.17-fold increase, while efficiency decreases from approximately 0.13 to 0.09 (30.8% reduction). A further increase from 0.73 to 1.5 raises normalized power from approximately 0.25 to 0.75 (3-fold increase) but causes a further approximately 33.3% reduction in efficiency. Thus, $K_{r}$ = 0.73 represents a balanced operating point within the investigated discrete levels rather than a universal optimum.

Increasing the stiffness ratio enhances the transmission of deformation from the suspension system to the series-connected piezoelectric stack, thereby increasing the relative displacement $\left(X_{s}-X_{p}\right)$ responsible for electrical generation. As indicated by Equation (29), harvested power is directly proportional to the square of this relative deformation, which explains the observed increase in electrical output.

In contrast, increasing the mass ratio reduces the harvested power while slightly improving the conversion efficiency. A larger unsprung-to-sprung mass ratio increases the inertia of the coupled mechanical system and suppresses vibration transmission to the piezoelectric element. Consequently, the dynamic deformation of the stack decreases, reducing the available mechanical energy for conversion into electricity. These observations indicate that the stiffness ratio primarily governs strain-transfer effectiveness, whereas the mass ratio controls the inertial energy exchange within the suspension-harvester system.

To quantify the energy-flow effect of mass ratio, the Taguchi response data were further examined in terms of normalized harvested power. The mean normalized harvested power decreases from 35.00 at $M_{r}$ = 0.5 to 4.11 and 2.28 at $M_{r}$ = 2 and 8, respectively, corresponding to reductions of 88.3% and 93.5%. This trend is consistent with progressively reduced vibration-to-electrical energy transfer as the unsprung-to-sprung mass ratio increases. The ANOVA contribution of $M_{r}$ is 14.04% for the undamped and 12.50% for the damped model.

Dimensionless power and efficiency are used for parameter-sensitivity analysis, while dimensional quantities represent practical output. For a fixed reference system, $P_{0}$ remains constant and preserves the relative parameter trends.

### 5.2. Numerical Verification of the Electromechanical Model

The accuracy of the proposed analytical model was assessed through comparison with independently implemented Simulink simulations, as shown in Figure 6. Both approaches predict identical dynamic characteristics, including the primary suspension-bounce resonance near 1.45 Hz and the wheel-hop resonance in the vicinity of 9–10 Hz. The maximum difference between the two approaches remained below 8% over the investigated 0–15 Hz frequency range.

For an additional quantitative resonance check, the mechanical eigenvalues relation $det(K-\omega^{2}M)$ = 0, using the reference parameters in Table 1, gives the suspension-bounce and wheel-hop frequencies as 1.450 Hz and 9.961 Hz, respectively. These values agree closely with the resonance locations observed in Figure 6 (≈1.45 Hz and 9.96–10 Hz), with frequency differences of only 0.02% and 0.01%, respectively. The lower-frequency bounce mode governs the dominant harvested-power response, whereas the wheel-hop mode is primarily a mechanical response characteristic. Over the investigated frequency range, the analytical-Simulink deviation remains below 8%.

The close agreement confirms that the coupled dimensionless formulation successfully captures the interaction between vehicle suspension dynamics and piezoelectric energy generation. Moreover, the comparison demonstrates that the analytical framework can accurately predict displacement response, generated voltage, and harvested power over a broad frequency spectrum. The verified model therefore provides a reliable basis for the subsequent parametric and optimization studies.

For the adopted parameters, the series equivalent stiffness is approximately 25.99 kN/m compared with 26 kN/m for the baseline suspension, corresponding to only about 0.04% reduction in effective stiffness. The corresponding change in the lower suspension-bounce natural frequency is approximately 0.02%.

### 5.3. Influence of Excitation Amplitude and Frequency

The effects of excitation acceleration and frequency on the electrical response are presented in Figure 7. Increasing the excitation level from 0.25 g to 1 g produces an approximately proportional increase in both generated voltage and harvested power. This behavior is expected because larger excitation amplitudes induce greater suspension displacements and higher piezoelectric strain levels.

The frequency-response characteristics reveal that the maximum electrical output occurs near the suspension-bounce resonance. At resonance, dynamic amplification substantially increases the relative deformation of the piezoelectric stack, resulting in enhanced charge generation and voltage output. Since harvested power is proportional to the square of the generated voltage, the resonance condition yields the highest power density. These findings highlight the importance of tuning suspension-based energy harvesters to operate near dominant vehicle vibration modes.

For the reference excitation (A = 1 g, f = 1.45 Hz, and $R_{L}$ = 10 kΩ), the dimensional response predicts a peak voltage of approximately 37.03 V and harvested power of 137.13 mW from the analytical model, while the corresponding Simulink results are approximately 38.43 V and 147 mW.

The corresponding equivalent load current is approximately 4 mA, obtained from I = P/V. Compared with the baseline suspension, the series-connected configuration changes the effective stiffness from 26 to 25.99 kN/m, resulting in an estimated suspension natural-frequency change of only about 0.02%. Thus, within the adopted linear model, the small changes in effective stiffness indicate a negligible modification of the lower suspension-bounce frequency, while the configuration provides approximately 37.03–38.43 V and 137.13–147 mW of electrical output.

### 5.4. Effect of Electrical Load and Damping Parameters

Figure 8 shows the load-resistance and tyre-damping effects. The load-resistance response is evaluated over a wider resistance range to expose the theoretical impedance-matching peak, while the Taguchi analysis retains the investigated 1–100 kΩ resistance levels. The generated voltage increases with load resistance, whereas harvested power reaches a maximum at the impedance-matching condition.

For the reference bounce condition at f = 1.45 Hz, Equation (30) gives the optimum dimensionless load resistance as $R_{opt}^{*}$ = 1/Ω ≈ 1.10. Using $C_{p}$ = 60 nF, the corresponding dimensional optimum load resistance is $R_{out}$ ≈ 1.83 MΩ. This theoretical optimum lies above the 1–100 kΩ resistance investigated in the Taguchi analysis; therefore, the observed dominance of load resistance is interpreted only within the investigated range and is not considered a global optimum.

Tyre damping has only a minor influence on both voltage and power over the investigated range, making it a secondary parameter from the present energy-harvesting perspective.

### 5.5. Effect of Suspension Damping and Electromechanical Coupling

The influence of suspension damping and the electromechanical force factor is shown in Figure 9. Increasing suspension damping significantly reduces both voltage generation and harvested power. Physically, greater damping converts a larger portion of the vibration energy into heat before it can be transferred to the piezoelectric element. Since electrical generation depends on the relative deformation across the stack, any reduction in vibration amplitude directly diminishes harvesting performance.

Conversely, increasing the electromechanical force factor substantially improves the electrical response. A larger force factor strengthens the coupling between the mechanical and electrical domains, enabling more efficient conversion of mechanical energy into electrical energy. As evident from Equations (28) and (29), the generated voltage and harvested power increase with coupling strength, leading to higher overall harvesting effectiveness.

### 5.6. Effect of Piezoelectric Capacitance

The influence of piezoelectric-stack capacitance is presented in Figure 10. Compared with other parameters, capacitance exerts only a moderate effect on harvesting performance. Increasing capacitance primarily alters the electrical time constant of the harvesting circuit while leaving the mechanical response largely unchanged.

As a result, the generated voltage and harvested power vary only through the load-dependent electrical term in Equation (28). These findings indicate that capacitance is a secondary design parameter, whereas load resistance, stiffness ratio, and electromechanical coupling play considerably more dominant roles in determining overall harvester performance.

### 5.7. Influence of Mechanical Parameters on Voltage Frequency Response

The frequency-response analysis shown in Figure 11, Figure 12 and Figure 13 provides further insight into the interaction between suspension dynamics and energy harvesting.

Suspension damping suppresses the resonance peak more effectively than tyre damping because it acts directly on the relative motion responsible for piezoelectric deformation. Consequently, increasing suspension damping significantly decreases the voltage amplitude near resonance.

Variations in unsprung mass primarily affect the wheel-hop resonance, whereas changes in sprung mass shift the bounce resonance toward lower frequencies. These trends are consistent with classical vehicle dynamics and confirm that the coupled model preserves the fundamental behavior of the underlying suspension system. In contrast, changes in piezoelectric stack mass produce negligible influence because the stack mass is several orders of magnitude smaller than the sprung and unsprung masses.

Similarly, tyre stiffness mainly influences the wheel-hop mode, while suspension stiffness controls the bounce resonance frequency. The piezoelectric-stack stiffness has a limited effect on the overall response because the proposed series configuration produced only a small change in the effective suspension stiffness, as predicted by Equations (4) and (5). This observation confirms one of the principal advantages of the proposed harvester arrangement: energy harvesting can be achieved without significantly altering the dynamic characteristics of the suspension.

### 5.8. Combined Influence of Stiffness Ratio and Damping Ratio

Figure 14 further shows the combined effect of stiffness ratio and damping ratio. Harvested power increases with stiffness ratio but decreases with damping ratio, indicating a trade-off between deformation transfer and vibration dissipation.

### 5.9. Statistical Optimization Using Taguchi-ANOVA

The Taguchi response and ANOVA results are presented in Figure 15 and Table 2 and Table 3. Load resistance is the dominant factor for both undamped and damped systems, contributing approximately 63.60% and 58.48% of the total variation in harvested power, respectively. The stiffness ratio contributes 19.65% and 17.92%, while the mass ratio contributes 14.04% and 12.50% for the undamped and damped models, respectively. The remaining factors have comparatively smaller effects. These statistical results are consistent with the deterministic trends discussed in Section 5.1, Section 5.2, Section 5.3, Section 5.4, Section 5.5 and Section 5.6 and identify electrical loading and mechanical strain transfer as the primary design considerations.

A concise comparison of the baseline suspension, proposed series-connected configuration, parallel-PZT configuration, and the experimental benchmark from the literature is provided in Table 4.

## 6. Conclusions

This study developed a coupled electromechanical model of a quarter-car suspension incorporating a series-connected multilayer PZT-5H stack. The analytical model was independently verified using Simulink, with the primary suspension-bounce resonance occurring near 1.45 Hz and the analytical-numerical deviation remaining below 8%. At the reference excitation, the model predicts approximately 37.03–38.43 V and 137.13–147 mW of harvested electrical output.

The parametric analysis shows a clear power-efficiency trade-off: increasing the stiffness ratio from 0.2 to 1.5 increases normalized harvested power from approximately 0.06 to 0.75, while conversion efficiency decreases from approximately 0.13 to 0.06. Within the investigated parameter range, electrical load resistance is the dominant factor, contributing 63.60% and 58.48% of the harvested-power variation, followed by stiffness ratio (19.65% and 17.92%) and mass ratio (14.04% and 12.50%).

The present study is limited by the use of linear electromechanical modeling and idealized harmonic excitation, while the available experimental data serve as an external benchmark rather than direct full-scale validation. Future work will therefore focus on experimental validation of the proposed series-connected configuration under realistic, standard road-roughness excitation (e.g., an ISO 8608 class-based random profile [36]), a dedicated 3-D COMSOL finite-element electromechanical model for cross-verification, quantitative error metrics (RMSE and mean percentage error) over the full frequency sweep, and the evaluation of practical power-management and energy storage circuits.

## Figures and Tables

**Figure 1:**
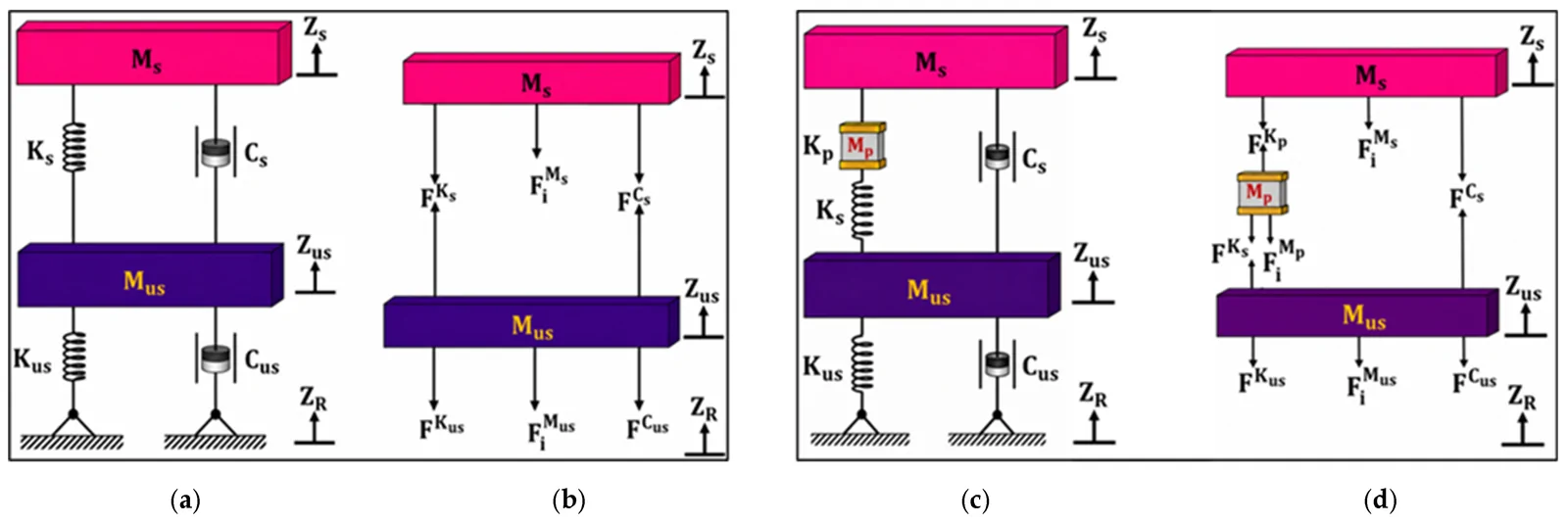
Quarter-car suspension configurations and corresponding free-body diagrams: (**a**) conventional suspension model without piezoelectric integration, (**b**) conventional suspension FBD, (**c**) series-connected PZT-5H suspension model, and (**d**) corresponding FBD.

**Figure 2:**
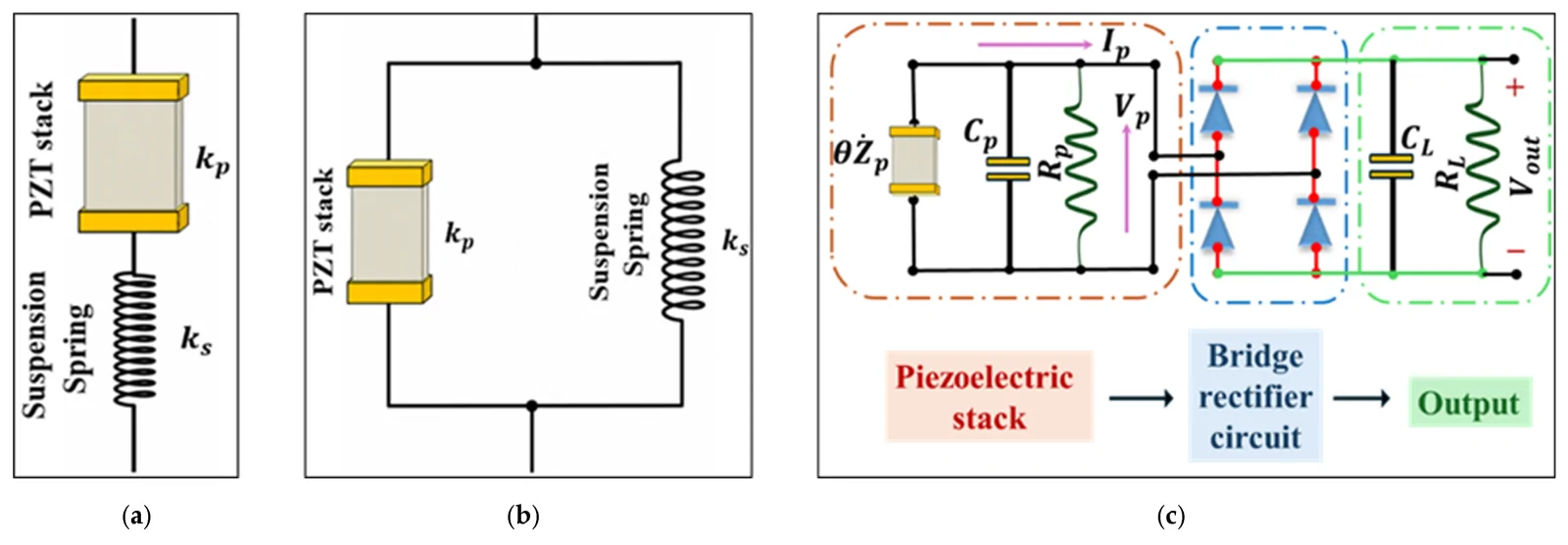
Comparison of the proposed series-connected and conventional parallel-connected PZT-5H configurations integrated with the vehicle suspension spring: (**a**) series-connected configuration, (**b**) parallel-connected configuration, and (**c**) equivalent electrical circuit with full-wave bridge rectification and resistive load.

**Figure 3:**
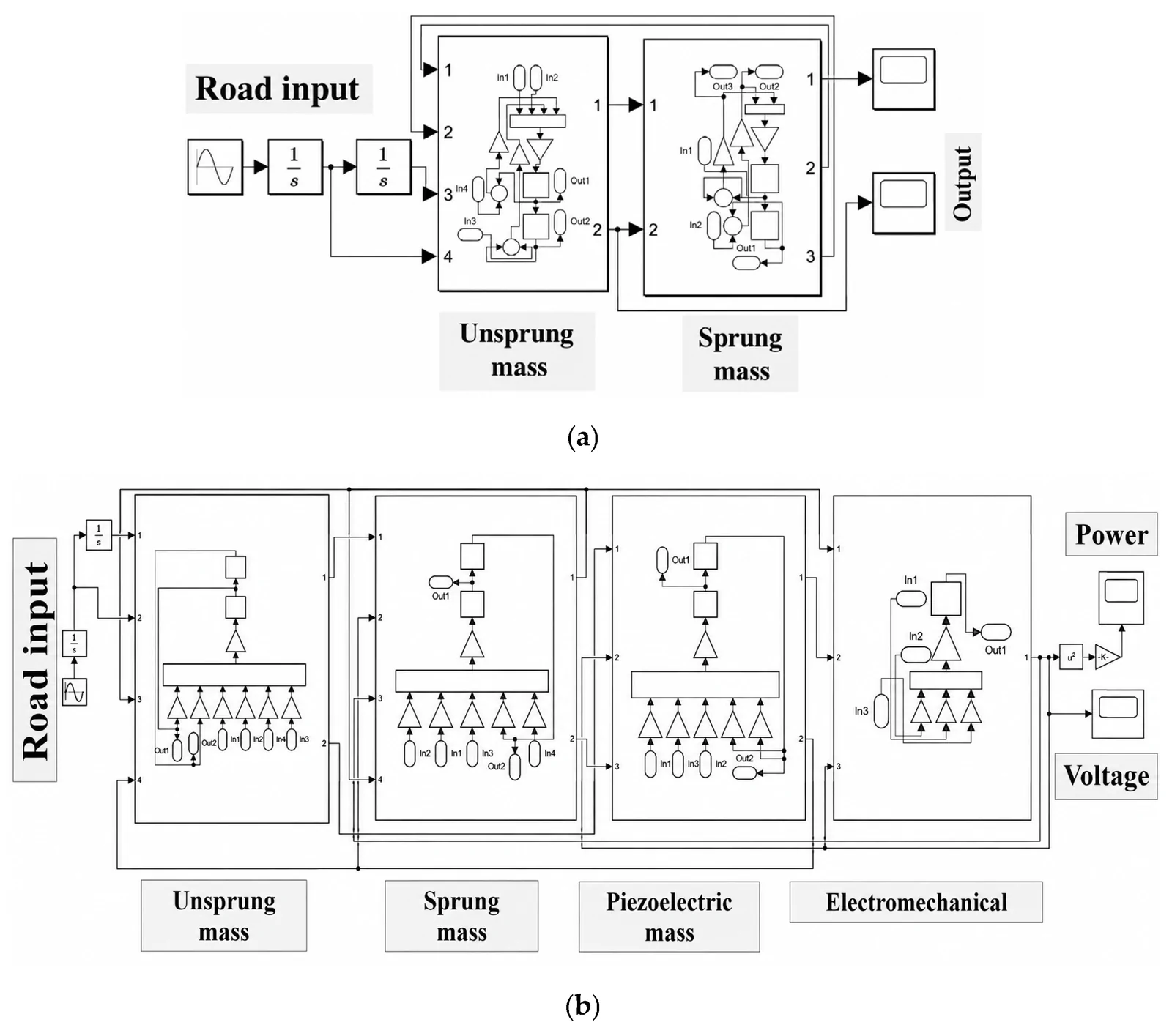
Simulink implementation of the (**a**) conventional quarter-car model and (**b**) proposed series-connected PZT electromechanical model.

**Figure 4:**
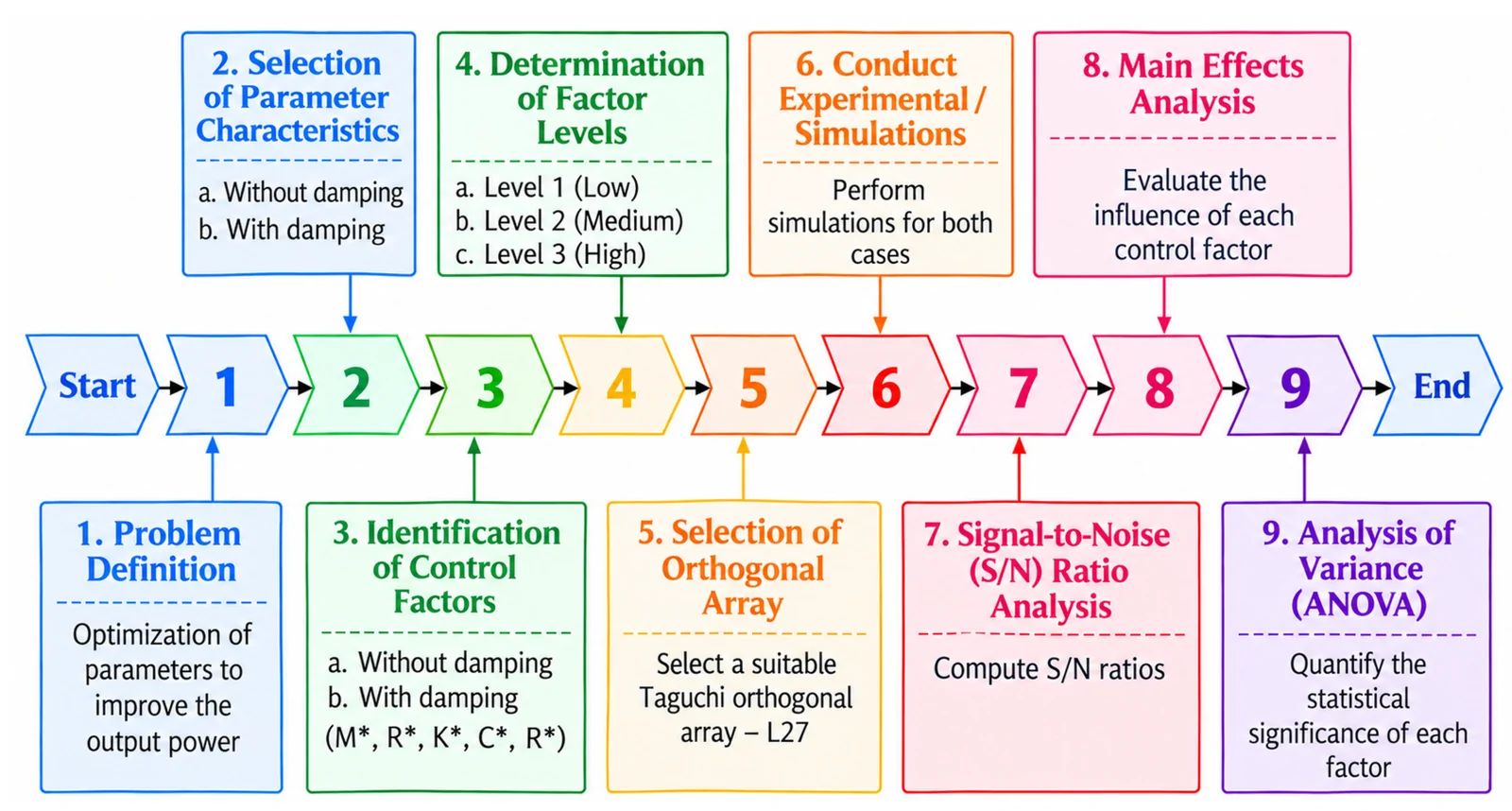
Process for Taguchi DOE: without damping and with damping.

**Figure 5:**
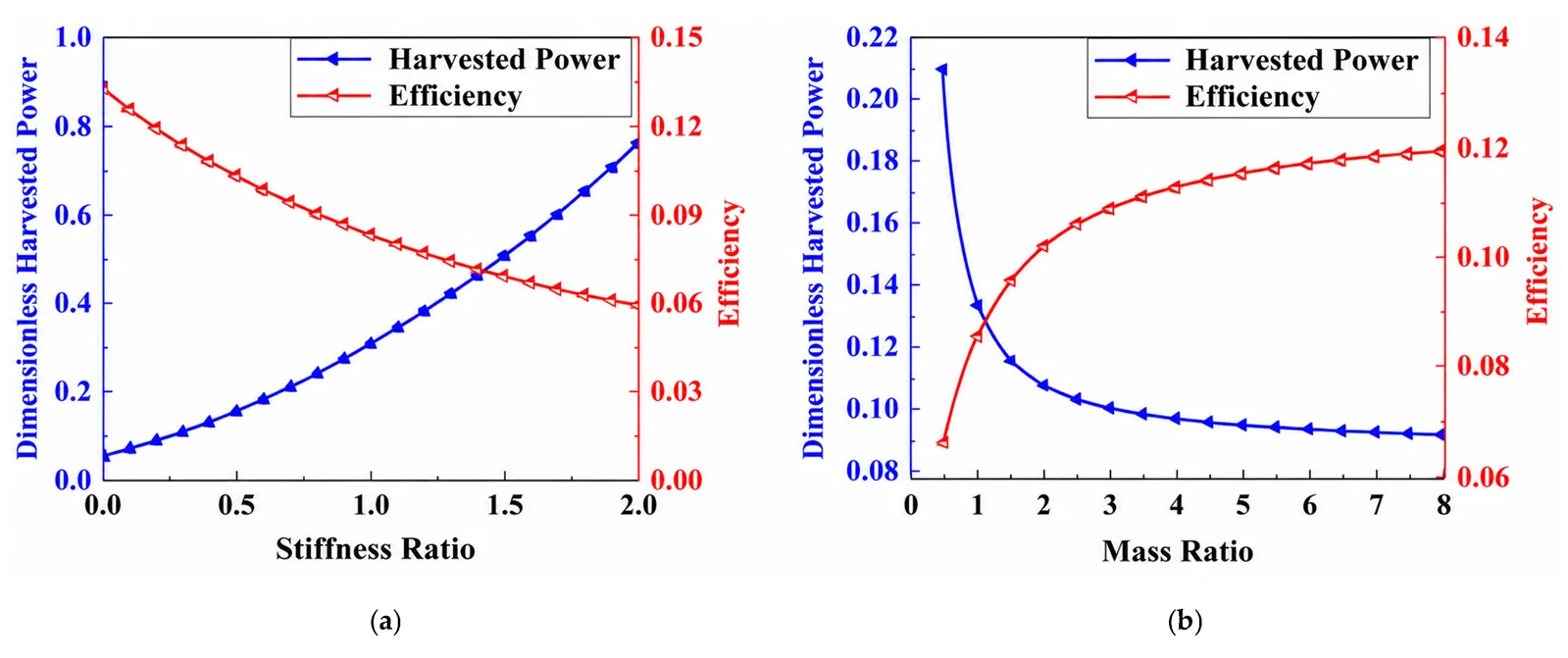
Effect of the dimensionless (**a**) stiffness ratio ($K_{r}$) and (**b**) mass ratio ($M_{r}$) on normalized harvested power and electromechanical conversion efficiency.

**Figure 6:**
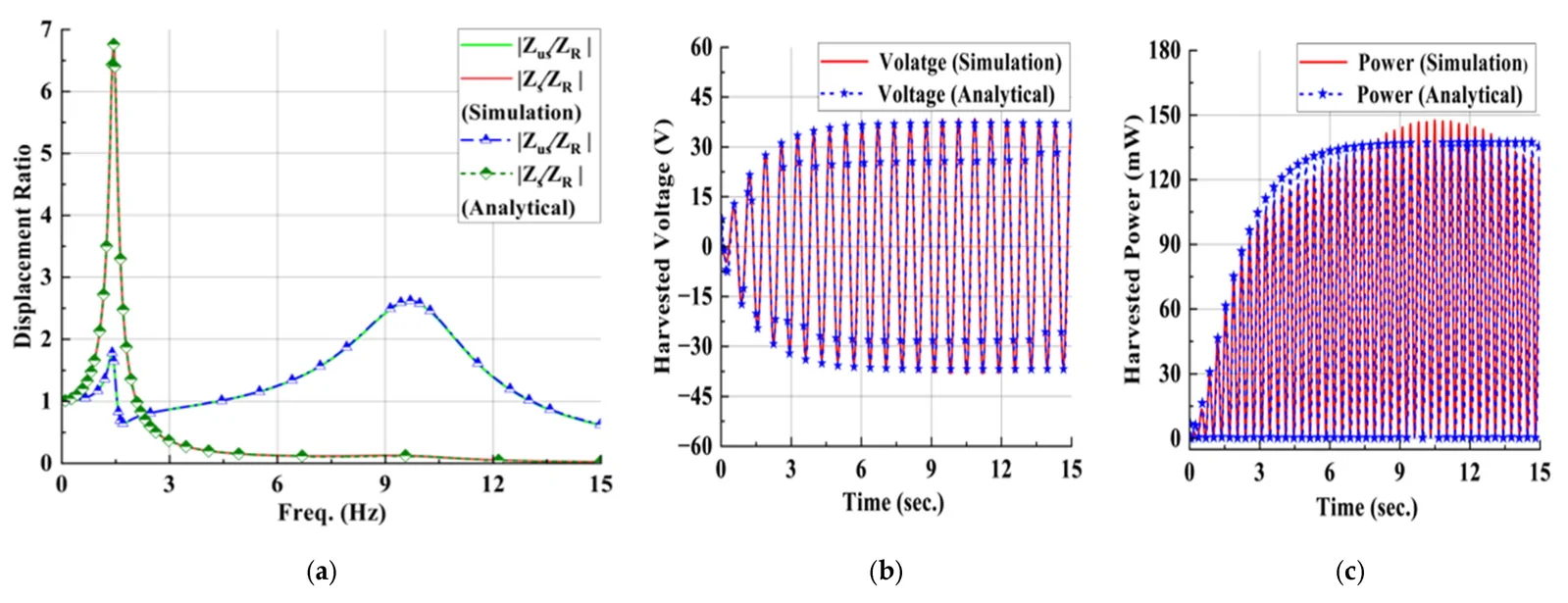
Analytical and Simulink verification of the proposed electromechanical model: (**a**) frequency response, (**b**) generated voltage, and (**c**) harvested power.

**Figure 7:**
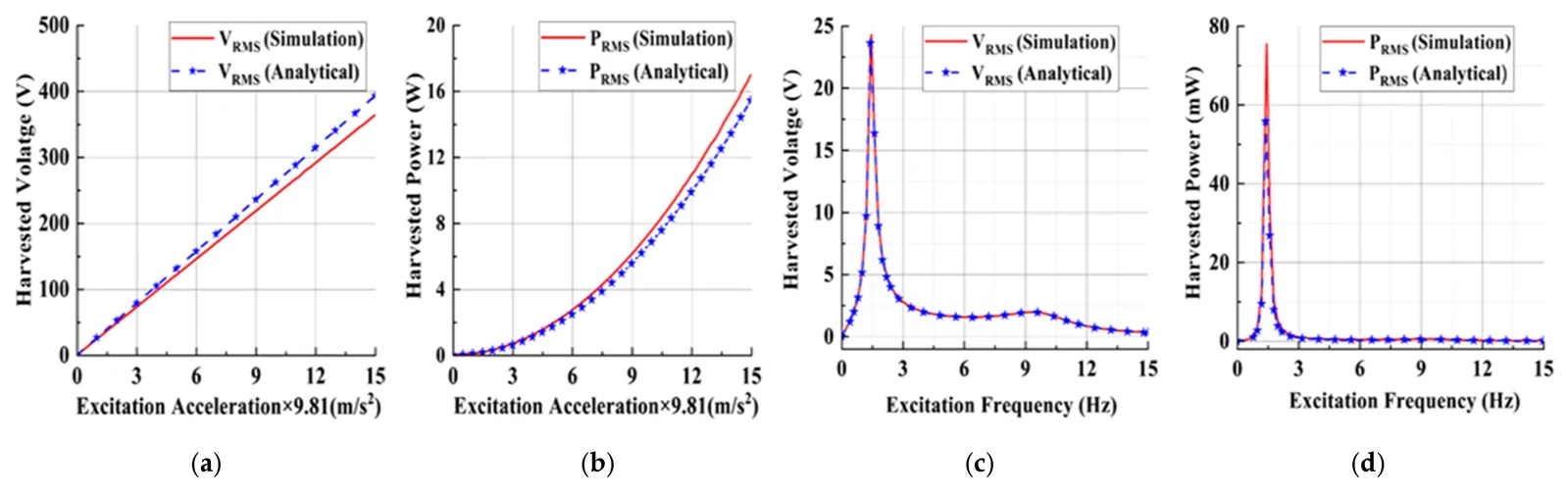
Effect of excitation acceleration and frequency on (**a**,**c**) generated voltage and (**b**,**d**) harvested power.

**Figure 8:**
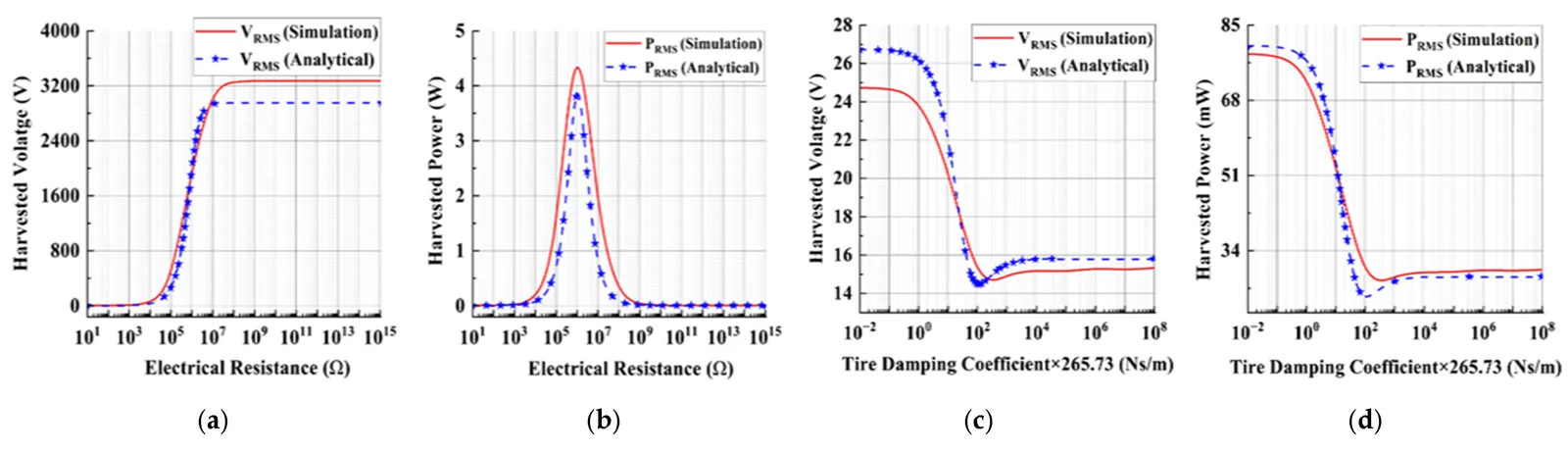
Effect of (**a**,**b**) load resistance and (**c**,**d**) tyre-damping coefficient on generated voltage and harvested power.

**Figure 9:**
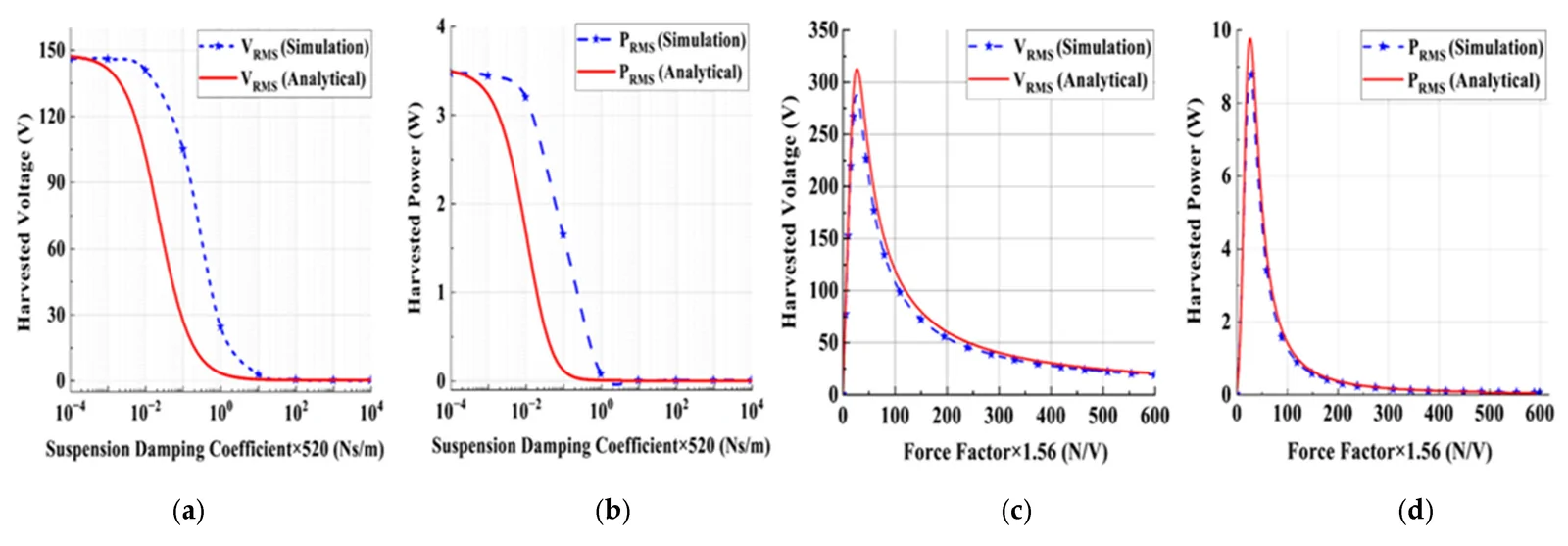
Effect of (**a**,**b**) suspension damping coefficient and (**c**,**d**) electromechanical force factor on generated voltage and power.

**Figure 10:**
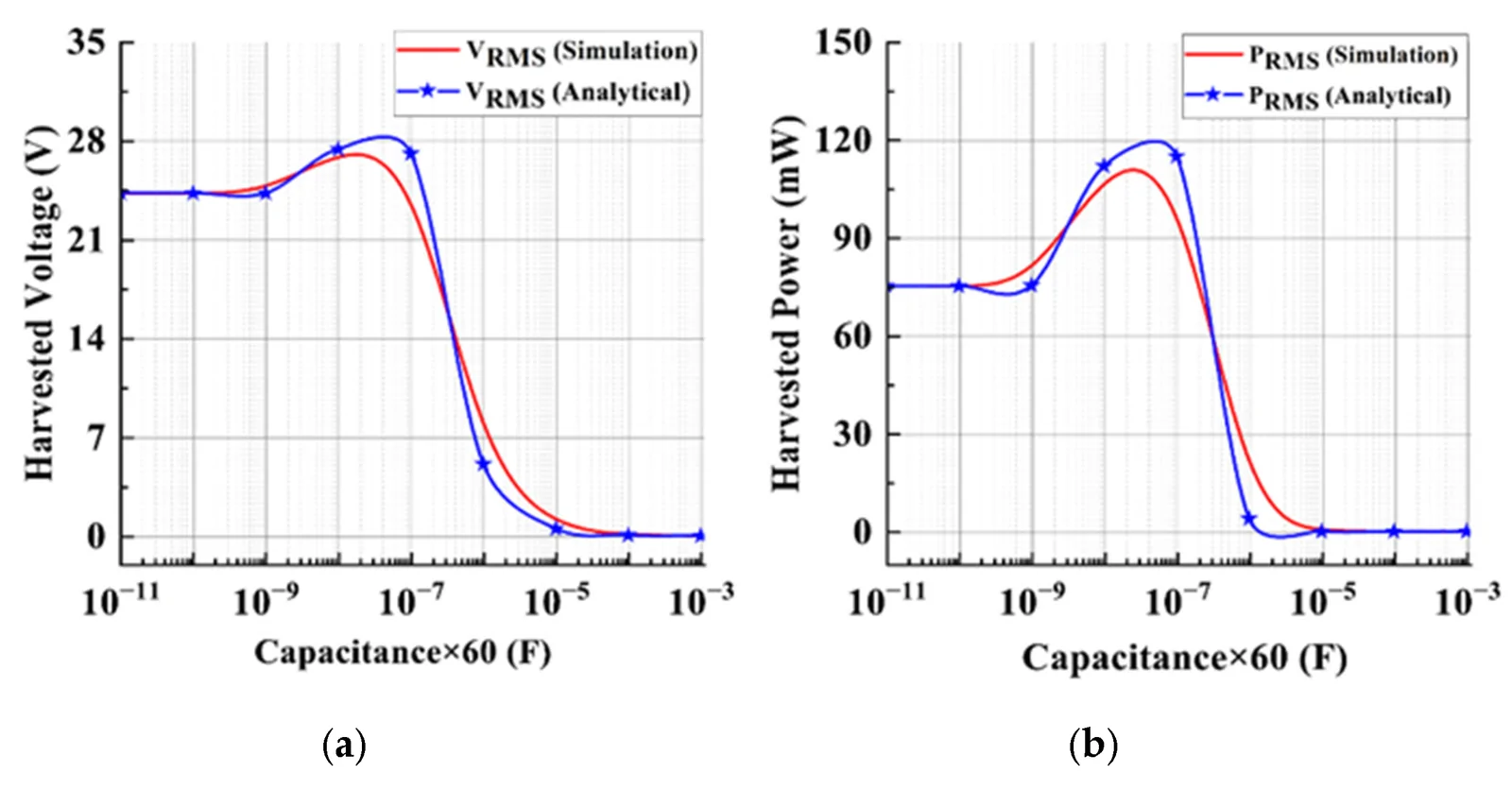
Effect of piezoelectric capacitance on (**a**) generated voltage and (**b**) power.

**Figure 11:**
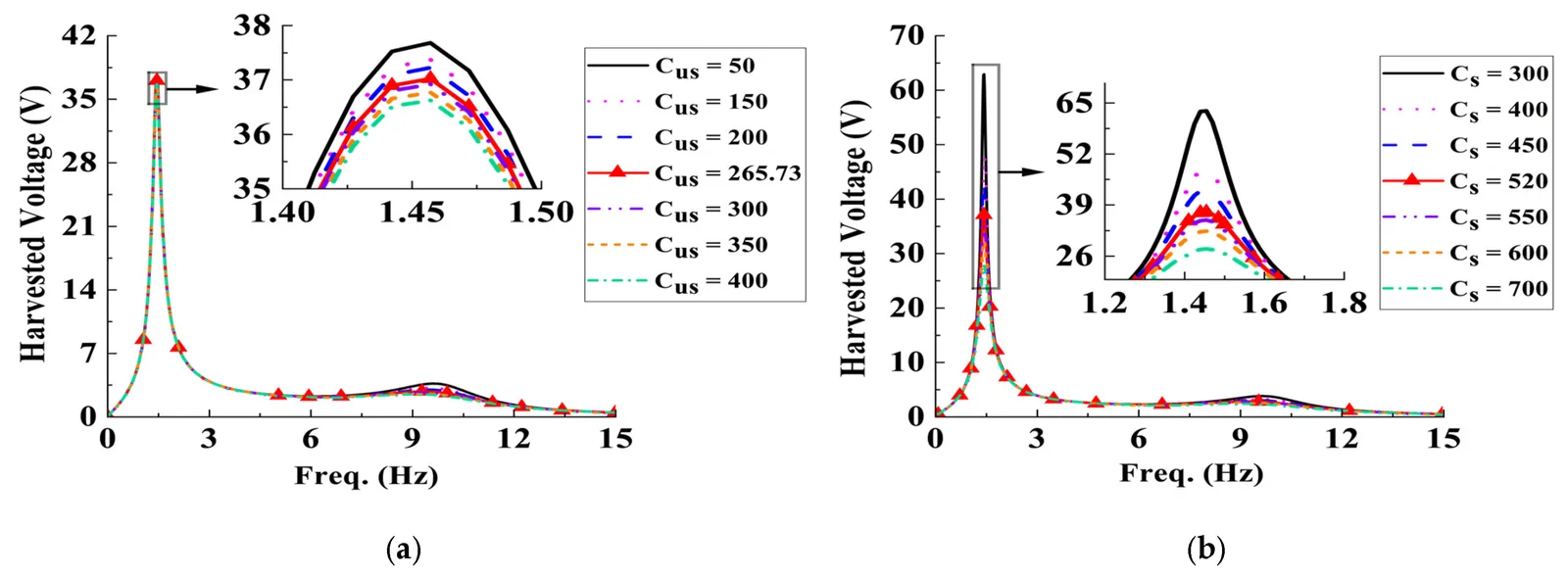
Frequency response of generated voltage for different (**a**) tyre-damping coefficients and (**b**) suspension damping coefficients.

**Figure 12:**
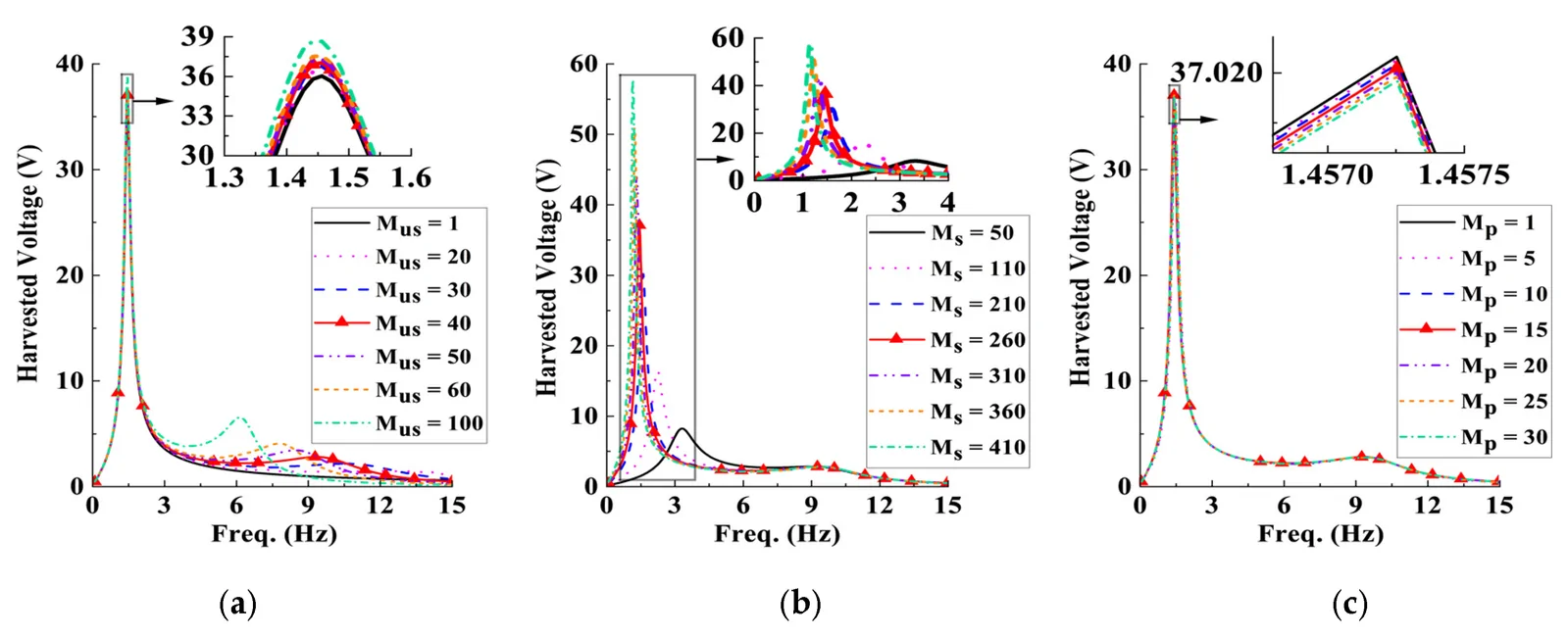
Frequency response of generated voltage for different (**a**) unsprung mass, (**b**) sprung mass, and (**c**) piezoelectric-stack masses.

**Figure 13:**
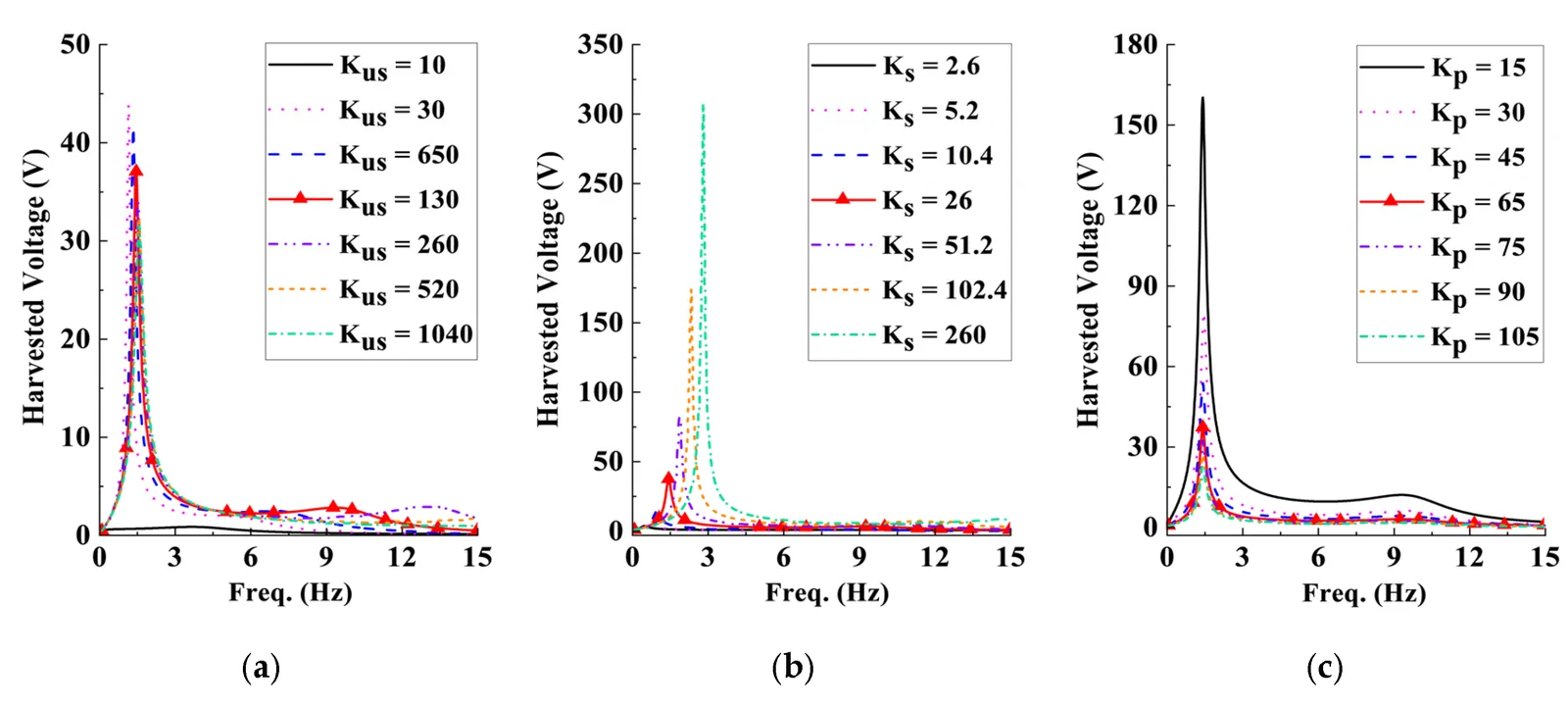
Frequency response of generated voltage for different (**a**) tyre stiffnesses, (**b**) suspension stiffnesses, and (**c**) piezoelectric-stack stiffnesses.

**Figure 14:**
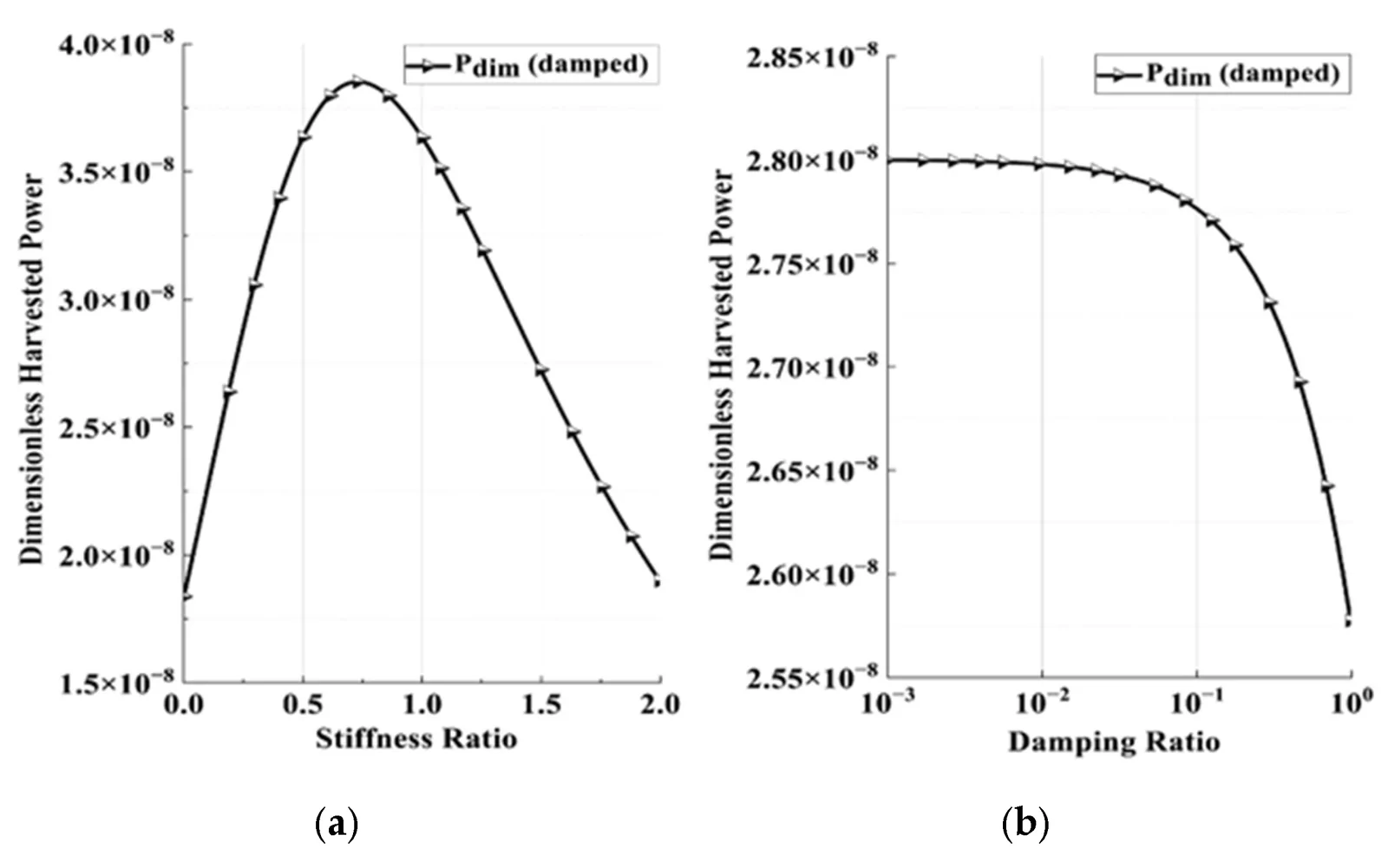
Effect of dimensionless (**a**) stiffness ratio and (**b**) damping ratio on normalized harvested power.

**Figure 15:**
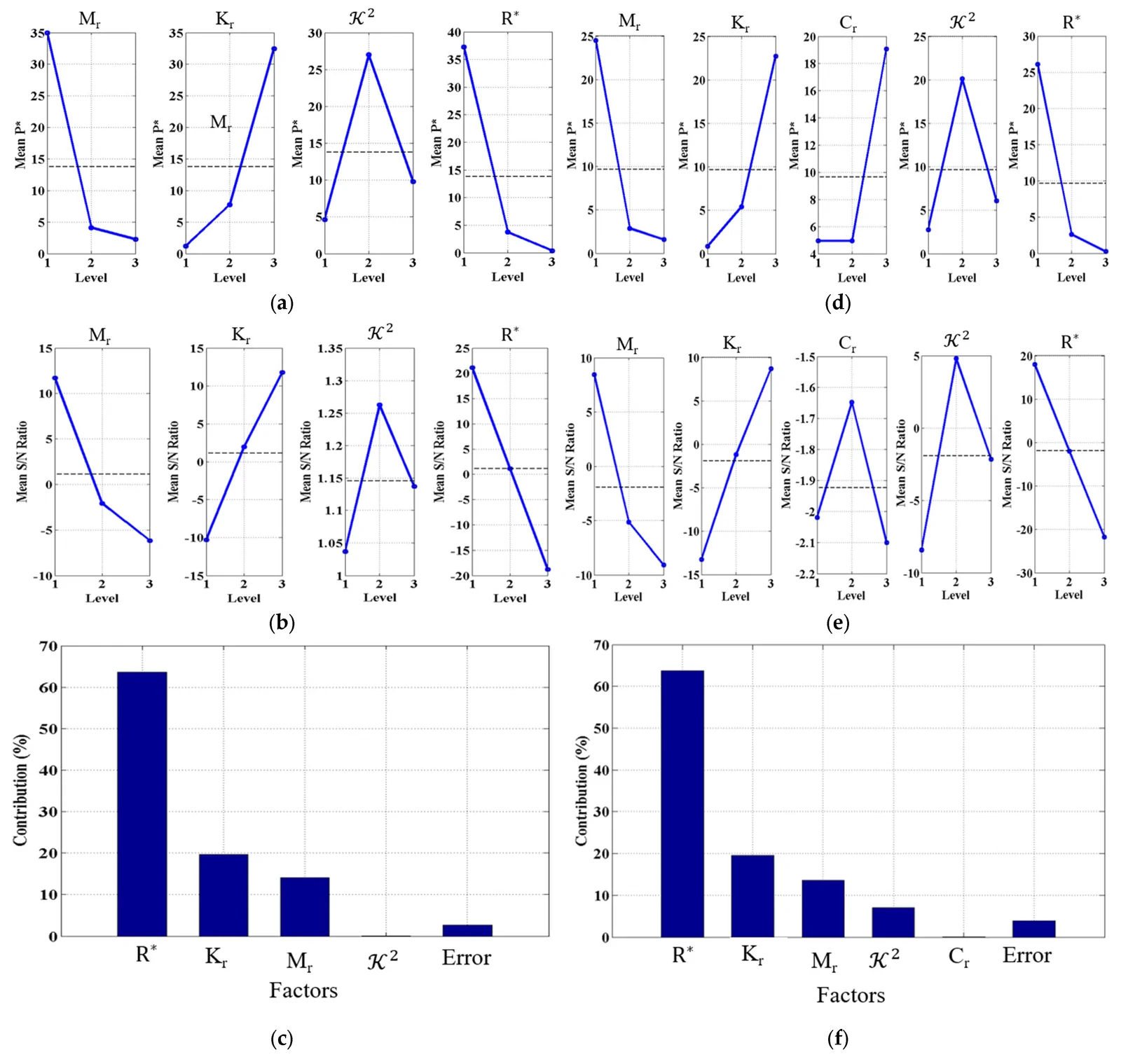
Taguchi–ANOVA optimization of dimensionless harvested power: (**a**,**d**) mean response, (**b**,**e**) S/N ratio, and (**c**,**f**) ANOVA contribution for undamped and damped systems.

**Table 1:** Quarter suspension model and piezoelectric parameters used in analysis.

Parameters and Acronyms	Values	Unit
Quarter vehicle mass ($M_{s}$)	260	kg
Unsprung mass ($M_{us}$)	40	kg
Piezoelectric mass ($M_{p}$)	0.015	kg
Suspension spring stiffness ($K_{s}$)	26	kN/m
Suspension damping coefficient ($C_{s}$)	520	N·s/m
Wheel-tyre stiffness ($K_{us}$)	130	kN/m
Wheel-tyre damping coefficient ($C_{us}$)	265.73	N·s/m
PZT stack stiffness ($K_{p}$)	65	MN/m
PZT blocked capacitance ($C_{p}$)	60	nF

**Table 2:** Taguchi response (undamped and damped).

	Undamped				Damped				
Factor	Level 1	Level 2	Level 3	∆ (Effect Range)	Level 1	Level 2	Level 3	∆ (Effect Range)	Rank
Dimensionless electrical load (R*)	21.11	1.13	−18.79	39.91	18.01	−1.98	−21.80	39.82	1
Dimensionless stiffness ratio ($K_{r}$)	−10.34	1.98	11.79	22.13	−13.28	−1.21	8.72	22.01	2
Dimensionless mass ratio ($M_{r}$)	11.71	−2.1	−6.18	17.89	8.46	−5.16	−9.07	17.53	3
Dimensionless electromechanical coupling factor $\left(K^{2}\right)$	1.04	1.26	1.14	0.23	−8.42	4.81	−2.15	13.23	4
Dimensionless damping ratio ($C_{r}$)	-	-	-	-	−2.02	−1.65	−2.10	0.45	5

**Table 3:** Standard ANOVA response (undamped and damped).

	Undamped					Damped				
Factors	Sum Sq.	D.F.	Mean Sq.	F	Cont. (%)	Sum Sq.	D.F.	Mean Sq.	F	Cont. (%)
$R^{*}$	7165.9	2	3583.0	211.10	63.60	7133.8	2	3566.9	100.94	58.48
$K_{r}$	2213.8	2	1106.9	65.22	19.65	2186	2	1093	30.93	17.92
$M_{r}$	1581.8	2	790.92	46.60	14.04	1524.7	2	762.34	21.57	12.50
$K^{2}$	0.229	2	0.115	0.007	0.002	788.22	2	394.11	11.15	6.46
$C_{r}$	-	-	-	-	-	1.0519	2	0.53	0.015	0.01
Error	305.51	18	16.97	-	2.7114	565.4	16	27.29	-	4.63
Total	11,267	26	-	-	100	12,199.17	26	-	-	100

**Table 4:** Baseline, proposed, parallel, and literature benchmark comparison.

Configuration	Electrical Output	Mechanical Effect	Basis
Baseline suspension	0 V, 0 W	$K_{s}$ = 26.00 kN/m	Present model
Proposed model	≈37.03–38.43 V, ≈137.13–147 mW	$K_{eq}$ = 25.99 kN/m ≈ $K_{s}$	Equations (4) and (5)
Parallel-PZT	Configuration dependent	$K_{eq}$ = 65.026 MN/m	Equation (4)
Experimental PZT suspension [11]	0.12 V, 0.36 mW at 0.05 g	Small-scale system	Darabseh et al. [11]

## Data Availability

The raw data supporting the conclusions of this article will be made available by the authors on request.

## Abbreviations

The following abbreviations are used in this manuscript:

QCM	Quarter-Car Model
PZT	Lead Zirconate Titanate
PVEH	Piezoelectric Vibration Energy Harvesting
DOE	Design of Experiments
ANOVA	Analysis of Variance
S/N	Signal-to-Noise Ratio
RMS	Root Mean Square

## Appendix A

### Appendix A.1

(A1)
$$(M_{s}s^{2}+C_{s}s+K_{p}{)Z}_{s}-C_{s}sZ_{us}-K_{p}Z_{p}+\theta V=0$$

The unsprung-mass equation becomes

(A2) $${-C}_{s}sZ_{us}+\left(M_{us}s^{2}+C_{s}s+C_{us}s+K_{p}+K_{us}\right)Z_{us}-K_{s}Z_{p}={(C}_{us}s+K_{us})Z_{R}$$

The piezoelectric-stack equation is

(A3) $$-K_{s}Z_{s}-K_{s}Z_{us}+(M_{p}s^{2}+K_{s}+K_{p}{)Z}_{p}-\theta V=0$$

The electrical equation is

(A4) $$-\theta sZ_{s}+\theta sZ_{p}+\left[C_{p}s+\frac{1}{R}\right]V=0$$

Solving Equation (A4) explicitly for the generated voltage gives

(A5) $$V(s)=\frac{\theta Rs(Z_{s}-Z_{p})}{1+RC_{p}s}$$

### Appendix A.2

Parameters for Taguchi and ANOVA analyses.

**Table A1:** Taguchi control factors and levels (undamped and damped).

	Undamped			Damped		
Control Parameter	Level 1 (Low)	Level 2 (Medium)	Level 3 (High)	Level 1 (Low)	Level 2 (Medium)	Level 3 (High)
Mass Ratio ($M_{r}$)	0.5	2.0	8.0	0.5	2.0	8.0
Stiffness Ratio ($K_{r}$)	0.2	0.73	1.5	0.2	0.73	1.5
Force Factor ($K^{2}$)	0.5θ	1θ	2θ	0.5θ	1θ	2θ
Load Resistance (R*, in kΩ)	1	10	100	1	10	100
Damping Ratio ($C_{r}$)	-	-	-	0.05	0.15	0.30

**Table A2:** Taguchi L27 orthogonal array (OA) (undamped and damped).

	Undamped						Damped						
Run	$M_{r}$	$K_{r}$	$K^{2}$	R*	$P_{out}^{*}$	S/N Ratio	$M_{r}$	$K_{r}$	$C_{r}$	$K^{2}$	R*	$P_{out}^{*}$	S/N Ratio
1	1	1	1	1	4.99	13.97	1	1	1	1	1	3.50	10.87
2	1	1	2	2	0.50	−6.03	1	1	2	2	2	0.35	−9.13
3	1	1	3	3	0.05	−26.03	1	1	3	3	3	0.03	−29.13
4	1	2	1	2	4.99	13.96	1	2	1	2	3	0.35	−9.13
5	1	2	2	3	0.50	−6.04	1	2	2	3	1	34.91	30.86
6	1	2	3	1	49.87	33.96	1	2	3	1	2	3.49	10.86
7	1	3	1	3	2.29	7.19	1	3	1	3	2	16.02	24.09
8	1	3	2	1	228.9	47.19	1	3	2	1	3	1.60	4.10
9	1	3	3	2	22.89	27.19	1	3	3	2	1	160.23	44.09
10	2	1	1	2	0.25	−11.99	2	1	1	2	3	0.02	−35.09
11	2	1	2	3	0.03	−31.99	2	1	2	3	1	1.76	4.91
12	2	1	3	1	2.51	8.01	2	1	3	1	2	0.18	−15.09
13	2	2	1	3	0.08	−22.02	2	2	1	3	2	0.55	−5.12
14	2	2	2	1	7.92	17.98	2	2	2	1	3	0.06	−25.12
15	2	2	3	2	0.79	−2.02	2	2	3	2	1	5.55	14.88
16	2	3	1	1	22.86	27.18	2	3	1	1	1	16.00	24.08
17	2	3	2	2	2.29	7.18	2	3	2	2	2	1.60	4.08
18	2	3	3	3	0.23	−12.82	2	3	3	3	3	0.16	−15.92
19	3	1	1	3	0.02	−33.31	3	1	1	3	2	0.15	−16.41
20	3	1	2	1	2.16	6.69	3	1	2	1	3	0.02	−36.41
21	3	1	3	2	0.22	−13.31	3	1	3	2	1	1.51	3.59
22	3	2	1	1	4.97	13.93	3	2	1	1	3	0.03	−29.16
23	3	2	2	2	0.50	−6.07	3	2	2	2	1	3.48	10.84
24	3	2	3	3	0.05	−26.07	3	2	3	3	2	0.35	−9.16
25	3	3	1	2	1.13	1.09	3	3	1	2	1	7.94	18.00
26	3	3	2	3	0.11	−18.91	3	3	2	3	2	0.79	−2.00
27	3	3	3	1	11.34	21.09	3	3	3	1	3	0.08	−22.00
